# An Acute, Rather Than Progressive, Increase in Temperature-Humidity Index Has Severe Effects on Mortality in Laying Hens

**DOI:** 10.3389/fvets.2020.568093

**Published:** 2020-11-05

**Authors:** Seokmin Kang, Da-Hye Kim, Sang Lee, Taehoon Lee, Kyung-Woo Lee, Hong-Hee Chang, Byunghern Moon, Tugay Ayasan, Yang-Ho Choi

**Affiliations:** ^1^Division of Applied Life Sciences (BK21 Plus Program), Gyeongsang National University, Jinju, South Korea; ^2^Department of Animal Science and Technology, Konkuk University, Seoul, South Korea; ^3^Department of Animal Science, Gyeongsang National University, Jinju, South Korea; ^4^Institute of Agriculture and Life Sciences, Gyeongsang National University, Jinju, South Korea; ^5^Celltech Co., Ltd., Chungbuk, South Korea; ^6^Kadirli Academy of Applied Science, Osmaniye Korkut Ata University, Osmaniye, Turkey

**Keywords:** heat stress, laying hens, mortality, panting, temperature-humidity index (THI)

## Abstract

This study was conducted to evaluate the effects of temperature-humidity index (THI) on the mortality and the panting rates in hens exposed to varying thermal environments. Hens were challenged with an acute elevation in THI in Experiment 1, where dry-bulb temperature and relative humidity were set at ~27°C and 56% at the beginning of the experiment and changed to 36°C and 45% at its conclusion, respectively. In Experiment 2, different groups of hens were exposed to a progressive increase in THI, with similar ranges to those used in the previous experiment. In Experiment 3, the hens used in Experiment 2 were again challenged by THI conditions, the intensity of which ranged between those used in the previous two experiments. In Experiment 4, panting rates were recorded under varying THI. In the last, plasma biochemical profiles were determined in blood taken from hens subjected to experimental conditions similar to those in Experiment 2. When THI was acutely elevated from 24.2° to 32.1°C within 1 h and then maintained over 4.5 h, no mortality was detected in the first hour, but exceeded 95% after 5 h, and reached 100% at 5.5 h. A gradual increase in THI to 31.2°C over 6 h did not result in mortality during the first 3 h. When THI was set below the conditions in Experiment 1 but above those in Experiment 2, mortality was 29% at 4 h, 75% at 5 h, and 79% at 8 h. However, no mortality was detected in their respective control groups. Panting was not observed under 25.3°C and was largely variable under 30°C. However, all hens exhibited panting exceeding 250 counts/min and 60% mortality at 34°C when heat stress continued for a duration of up to 280 min. In Experiment 5, high ambient THI resulted in significant reductions in plasma albumin, amylase and aspartate aminotransferase, compared with those in control group (*P* < 0.05). These results suggest that an acute elevation of THI has more severe effects on mortality in hens than gradual changes even when temperature and humidity are similar in both cases.

## Introduction

The effects of heat stress in poultry include reduced feed intake, body weight gain, and production performance along with increased mortality (1–4), which results in a loss of profits in poultry farms. These negative effects are accompanied by deterioration in meat quality (5), animal welfare (2, 6–8), and immune functions (3). Heat stress also markedly affects post-absorptive metabolism of nutrients, such as carbohydrates, lipids, and proteins, independent of reduced feed intake due to coordinated changes in fuel supply and its utilization by multiple tissues (9).

Poultry are vulnerable to high temperature environments since they are covered with feathers and do not have sweat glands. These two factors reduce the efficiency of heat exchange between the body and the environment that is normally observed in animals with sweat glands. Therefore, increasing their rate of respiration is one of the key ways by which birds maintain their body temperature under increasing environmental temperatures.

Panting can be defined as a physiological phenomenon wherein animals/birds take strenuous, quick, shallow breaths with open mouths resulting in evaporative loss of water which helps alleviate heat stress from the body caused by either high temperature or physical activity (10, 11). It was shown that respiratory frequency increases in hens with the increase of ambient temperature (12). Panting helps remove carbon dioxide from the body which reduces carbon dioxide and hydrogen ions but increases bicarbonate ions in the blood stream, leading to an increased blood pH (10, 13), a condition known as respiratory alkalosis. Severe alkalosis may be associated with negative effects on performance in broilers and laying hens, including reduced feed intake, and increased mortality.

Mortality is the most detrimental, apparent, and irreversible response in animals challenged with stressful environments, and is easily detectable and quantifiable on the spot. Mortality begins to rise when ambient temperature increases above 20°C (14), or when average daily maximum temperature exceeds approximately 19°C during transportation and lairage (8). In most cases, mortality has been reported in a total number at the end of the study rather than reporting it in a time-dependent manner (1, 3).

There are a variety of experimental settings in which the effects of heat stress have been studied. Since both temperature and humidity interact to affect thermal conditions with which animals are challenged (14, 15), it may not be easy to compare and interpret the results obtained from different experimental settings. Temperature-humidity index (THI) is an indicator that considers both temperature and relative humidity when the effects of heat stress are evaluated (16). THI has been presented in studies including laying hens (15–17), broilers (18–20), and quails (21, 22). Although many studies have used THI to express thermal conditions, there is a lack of information available that could be useful for comparing effects of the severity and the duration of heat stress on poultry. In this study, we determined the effects of different THI conditions on mortality and panting responses in laying hens.

## Materials and Methods

### Animals and Experimental Design

Seventy-week-old Hy-Line Brown laying hens were housed into two-tiered battery cages, with four hens in each cage, in a windowless commercial layer-farm setting with a 16-h light: 8-h dark lighting schedule. The lower and upper cages were 57 and 145 cm above the floor, respectively. As tier levels in multi-tier battery cage systems can lead to temperature variations within a hen house during hot weather, this difference in the tier levels contributed toward producing differential thermal environments for the current study. Both a commercial layer mash feed and water via nipples were provided *ad libitum* throughout the experiments. The hens had at least 2 weeks to acclimate to the experimental environment. This study was approved by Institutional Animal Care and Use Committee (KU19153).

Hens were exposed to varying ambient conditions where temperature was increased from 26.0° to 40.0°C, while relative humidity varied between 37 and 60%. Both temperature and relative humidity were measured at both tiers during thermal challenges. Because heat stress is a function of ambient temperature and relative humidity (15), both dry-bulb temperature (T_db_) and relative humidity were measured. Wet-bulb temperature (T_wb_) was estimated using the method suggested by Stull (23). THI was then calculated from the following equation suggested by Zulovich and DeShazer (16):

$$\begin{array}{l} {\text{THI}}_{\text{layers}}=0.6{\text{T}}_{\text{db}}+\;0.4{\text{T}}_{\text{wb}} \end{array}$$

Where THI = temperature-humidity index (°C); T_db_ = dry-bulb temperature (°C); and T_wb_ = wet-bulb temperature (°C).

Four experiments were performed based on varying ambient temperature and relative humidity. All experiments were performed separately on different days, and the same hens were used in the Experiments 2 and 3. In order to minimize disturbance to the birds, the same number of experimenters throughout all experiments measured mortality at the set times. In Experiment 1, the effects of acute elevation in THI on mortality were determined. One group of hens (*n* = 24) were used as treatment group and another (*n* = 12) served as control. Heat blowers were set to increase the ambient temperature from 26° to 36°C within 1 h, and this temperature was maintained over the next 4.5 h, while mortality was observed. The temperature and relative humidity values recorded were 26°C and 56% at the beginning of the experiment group, and 36°C and 45.5% for the treatment and 30°C and 45% for the control at the conclusion, respectively. Mortality rates were determined at 0, 1, 5, and 5.5 h after the experiment began. Artificial ventilators and fans were turned off during the experiment.

Experiment 2 was performed for 9 h to measure the effects of a gradual increase (an about 1°C increase per hour) in THI on the mortality in birds. Mortality was observed every hour after the start of the experiment. One group of hens (*n* = 24) were used as treatment group and another (*n* = 12) served as control. The experimental conditions were similar to those in Experimental 1, as previously mentioned. The temperature and relative humidity values recorded were 27.0°C and 55.0% at the beginning of the 9-h experiment, and 35.5°C and 37.0% for the treatment group and 30.3°C and 39.0% for the control at its conclusion, respectively.

In Experiment 3, the effects of gradual, but high elevation (~2°C per hour) in THI on mortality were determined for 8 h in the hens that were previously used in Experiment 2. Mortality was observed every hour after the experiment began. One group of hens (*n* = 24) were used as treatment group and another (*n* = 12) served as control. The experimental conditions were similar to those in Experiments 1 and 2, and the THI was set below the parameters in Experiment 1 but above those in Experiment 2. The temperature and relative humidity values recorded for the treatment group were 28.0°C and 55.0% at the beginning of the experiment. They were 39.2°C and 39.5% for the treatment group and 32.5°C and 40.0% for the control at its conclusion, respectively.

In Experiment 4, the effects of different THI (°C) on panting (counts/min) were determined in laying hens exposed to varying THI for different time periods. Five groups of hens (*n* = 12 each group) were challenged with one of the five-THI conditions (25.3, 28.8, 30.0, 34.3, and 33.6°C) for different time periods (0, 40, 100, 160, or 280 min, respectively), and the hens' behavior was recorded for 10 min using video cameras beginning from the time points specified above. Five hens from each group were chosen that clearly demonstrated their behavior for the panting analysis. When the respiration rate exceeded 60/min with the mouth open, the behavior was considered panting (24). Mortality was also recorded during these periods.

In Experiment 5, hens were randomly divided into two groups of 8 birds and placed in experimental conditions similar to those in Experiment 2. In the treatment group, THI began to increase from 24.2° to 31.1°C over 5 h and was maintained for the next 3 h, whereas in the control group, THI remained between 24.4° and 26.6°C throughout the experiment. At the end of the 8-h experiment, hens were euthanized in a carbon dioxide chamber, and blood was then drawn into vacutainers containing heparin (#367874, BD Co., Ltd., Franklin Lakes, NJ, USA) through a cardiac puncture and centrifuged at 2,000 × g at 4°C for 10 min. Plasma was harvested, aliquoted and stored at −20°C until later analysis. Blood biochemicals, including corticosterone, were determined according to the methods described by Kim et al. (25).

### Statistical Analysis

Statistical analysis was carried out with GLM procedure of the SAS (SAS, 2004). Panting rates were analyzed using one-way ANOVA, followed by a Duncan multiple-range test to compare means. Blood data were analyzed by a *t-*test. Experimental unit was cage. *P* < 0.05 was considered significant. The following statistical model was used to analyze the data:

$$\begin{array}{l} y_{ij}=\mu +T_{i}+\varepsilon_{ij} \end{array}$$

where *y*_*ij*_ = _*ij*_ observation; μ = overall average; *T*_*i*_ = treatment effect; and ε_*ij*_ = random error.

## Results

**Figure 1:**
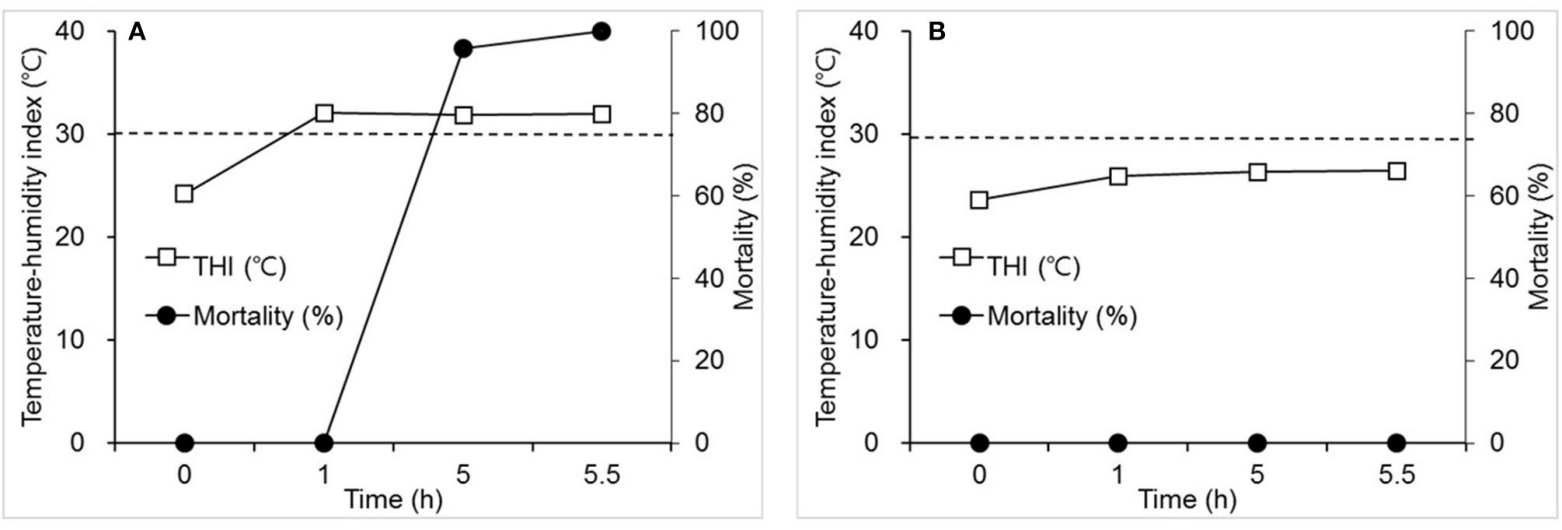
The effects of an acute elevation in temperature-humidity index (THI) (°C) on mortality (%) in laying hens. Hens were exposed to one of the two following THI conditions for 5.5 h. **(A)** THI: 24.2–32.0; dry-bulb temperature (Tdb) (°C): 26.8–36.0; web-bulb temperature (Twb) (°C): 20.3–26.6; and relative humidity (RH) (%): 56.0–45.5. **(B)** THI: 23.6–26.4; Tdb: 26.0–30.0; Twb: 20.0–21.5; and RH: 58.0–45.0. Mortality was measured at the time points specified (h). The dot line indicates THI of 30°C. *N* = 24 for A and 12 for B.

Figure 1 shows changes in THI and mortality over 5.5 h in Experiment 1. THI reached 32.1°C from 24.2°C in 1 h after the initiation of heat stress, and maintained plateau over the next 4.5 h (Figure 1A). Mortality was not detected during the first hour of heat stress, but there was more than 95% mortality in hens at 5 h, and 100% mortality at 5.5 h. However, mortality was not detected in the control group where THI increased from 23.6° to 26.0°C in 1 h after the initiation of heat stress (Figure 1B).

**Figure 2:**
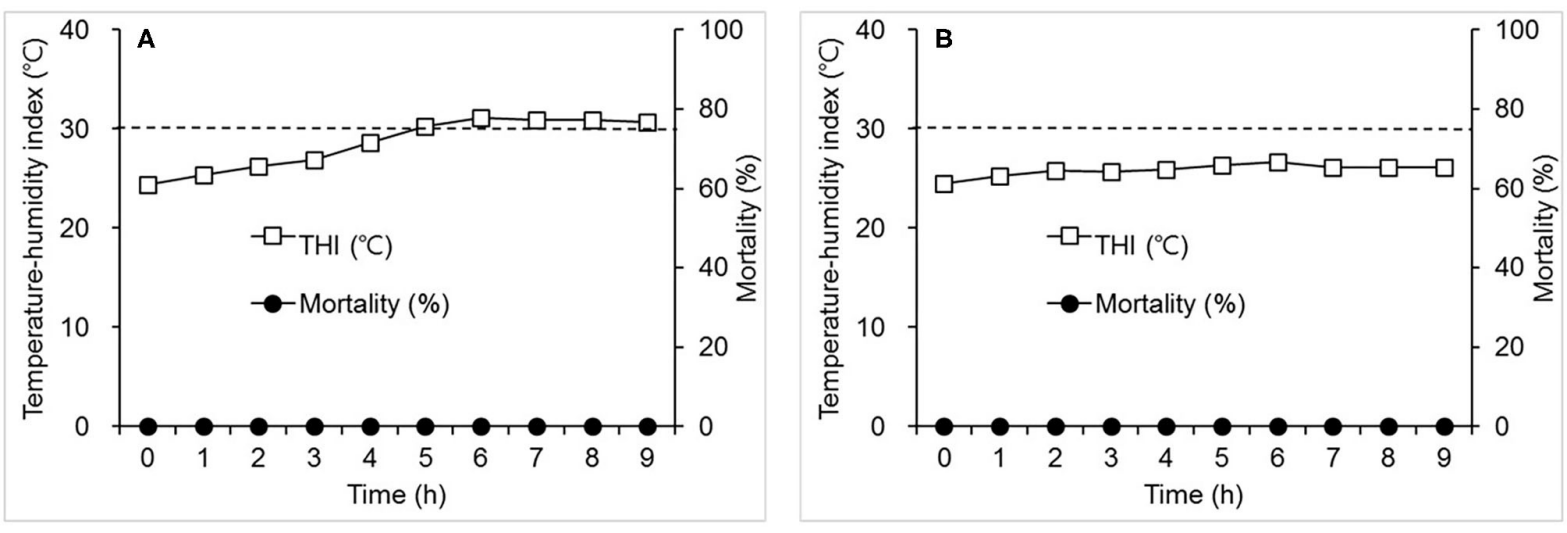
The effects of a moderate increase in temperature-humidity index (THI) (°C) on mortality (%) in laying hens. Hens were exposed to one of the two following THI conditions for 9 h. **(A)** THI: 24.3–31.2; dry-bulb temperature (Tdb) (°C): 27.0–35.5; web-bulb temperature (Twb) (°C): 20.4–25.3; and relative humidity (RH) (%): 55–37. **(B)** THI: 24.4–26.6; Tdb: 27.1–30.3; Twb: 20.5–21.3; and RH: 55.0–39.0. Mortality was measured at the time points specified (h). The dot line indicates THI of 30°C. *N* = 24 for A and 12 for B.

No mortality was detected in the hens from the treatment group in Experiment 2 that were exposed to an ambient environment with THI of 31.2°C which was achieved over 6 h after the initiation of heat stress (THI was 24.3°C at 0 h) and maintained for the next 3 h (Figure 2A). Control groups showed no mortality when THI was slightly elevated from 24.4° to 26.6°C in 6 h (Figure 2B).

**Figure 3:**
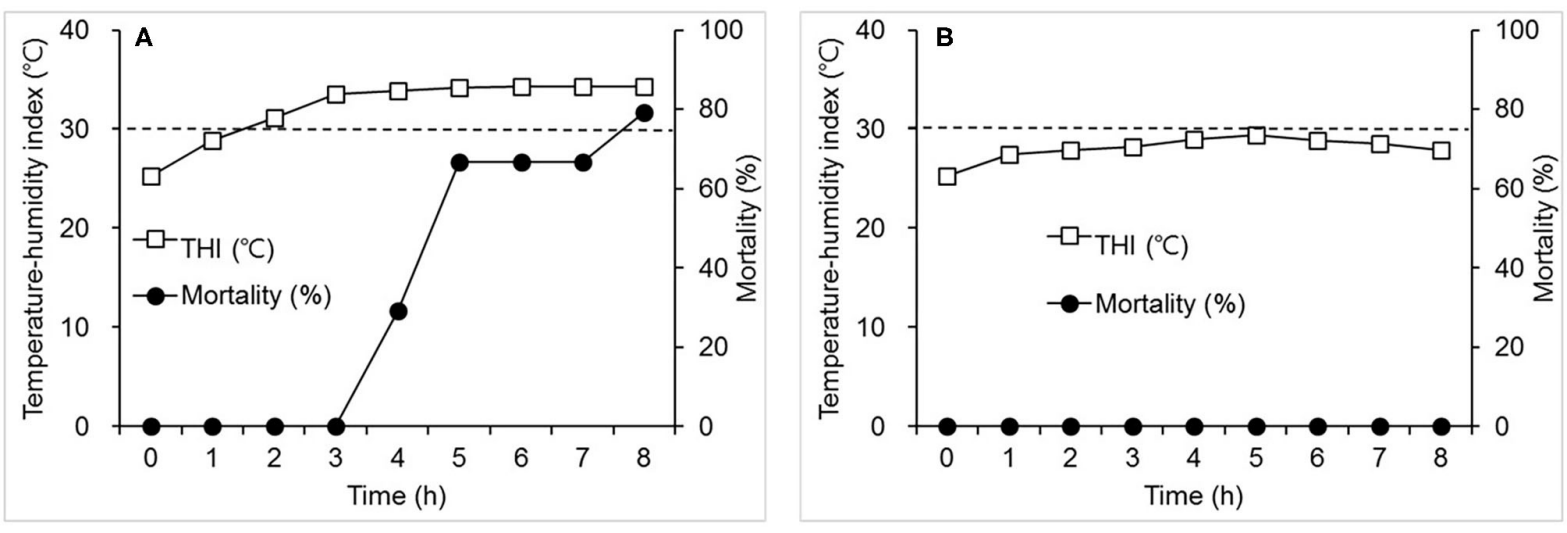
The effects of a moderate increase in temperature-humidity index (THI) (°C) on mortality (%) in the laying hens used in Experiment 2. Hens were exposed to one of the two following THI conditions for 8 h. **(A)** THI: 25.2–34.3; dry-bulb temperature (Tdb) (°C): 28.0–39.2; web-bulb temperature (Twb) (°C): 21.2–27.6; and relative humidity (RH) (%): 55.0–39.5. **(B)** THI: 25.2–29.4; Tdb: 28.0–32.5; Twb: 21.2–24.1; and RH: 55.0–40.0. Mortality was measured at the time points specified (h). The dot line indicates THI of 30°C. *N* = 24 for A and 12 for B.

Figure 3 shows the effects of THI on mortality in the hens exposed repeatedly to heat stress. THI was rapidly increased from 25.2° to 34.3°C within 3 h and maintained at a plateau over the next 5 h. Mortality was first detected at 4 h after the beginning of the thermal manipulation, 75% mortality was observed at 5 h, and 79% at 8 h (Figure 3A). No mortality was observed in the birds of the control group exposed to mild changes in THI up to 29.4°C for 5 h (Figure 3B).

**Figure 4:**
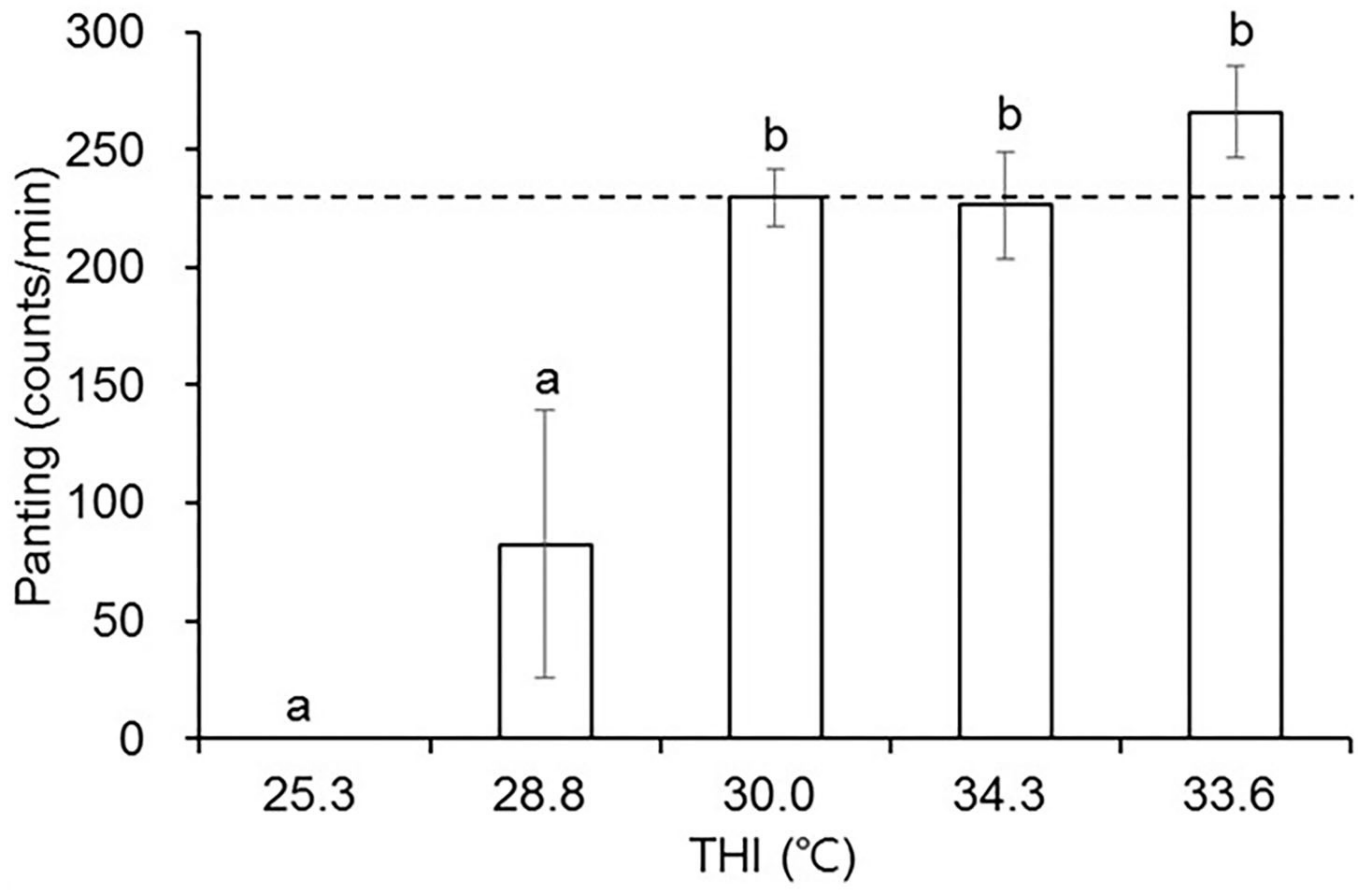
The effects of temperature-humidity index (THI) (°C) on panting (counts/min) in laying hens. Five groups (*n* = 12 each) of hens were exposed to one of the following five-THI conditions for different time periods (0, 40, 100, 160, or 280 min), and hens' behaviors were recorded and analyzed for panting frequency which was counted at the time points specified (min) (*n* = 5 each group). Temperature-humidity index (THI) (°C), dry-bulb temperature (°C), web-bulb temperature (°C), and relative humidity (%) were 25.3, 28.0, 55.0, and 21.2 for 0 min; 28.8, 32.1, 50.0, and 23.7 for 40 min; 30.0, 33.1, 54.0, and 25.4 for 100 min; 34.3, 38.4, 46.0, and 28.0 for 160 min; 33.6, 38.3, 40.0, and 26.5 for 280 min, respectively. The dot line indicates THI of 30°C. ^a,b^ Means with different superscripts differ significantly in panting frequency at *p* < 0.05.

Panting was not observed at 0 min when THI was at 25.3°C (Figure 4). Panting responses were largely variable at 30°C; 40% of the hens panted at 29°C. When THI was above 30°C, however, all of the hens showed panting-like behavior, exceeding 200 counts/min. Panting rates were over 250 counts/min with 60% mortality at 280 min when THI was elevated to and maintained at around 34°C. Although THI was similar, a longer exposure time to the thermal environment resulted in higher panting rates and mortality in laying hens (Figure 4).

No dead hens were found in Experiment 5. The high ambient THI resulted in significant reductions in albumin (10.6%), amylase (30.2%), and aspartate aminotransferase (AST) (23.3%), compared with those in control group (*P* < 0.05) (Table 1). No significant differences were found in the remaining parameters measured, including corticosterone.

**Table 1:** Effects of a moderate increase in temperature-humidity index (THI) on blood biochemicals in laying hens.

**Parameters**	**Control**	**Heat stress**
Albumin (g/dL)	2.08 ± 0.08	1.86 ± 0.07^*^
Amylase (U/L)	291 ± 21.3	203 ± 11.5^*^
Aspartate aminotransferase (U/L)	206 ± 17.9	158 ± 10.3^*^
Ca (mg/dL)	15.6 ± 0.36	15.9 ± 0.08
Cholesterol (mg/dL)	113 ± 16.1	100 ± 7.35
Creatine kinase (U/L)	1,723 ± 91.0	1,730 ± 93.5
Creatinine (mg/dL)	0.11 ± 0.01	0.11 ± 0.03
Globulin (g/dL)	4.00 ± 0.13	3.61 ± 0.17
Glucose (mg/dL)	205 ± 15.0	191 ± 16.3
Inorganic phosphate (mg/dL)	9.13 ± 0.57	9.50 ± 1.09
NH_3_ (umol/L)	796 ± 58.6	799 ± 30.4
Total protein (g/dL)	6.08 ± 0.20	5.53 ± 0.25
Triglycerides (mg/dL)	1,710 ± 251	1,254 ± 167
Uric acid (mg/dL)	8.10 ± 1.15	8.19 ± 0.90
Corticosterone (ng/ml)	23.2 ± 7.28	16.0 ± 2.15

* *p < 0.05*.

*Data show mean ± SEM (n = 8 each group)*.

## Discussion

Responses of hens to heat stress are different depending upon the severity of THI. When the increment rate of THI, beginning from 24°C, increased by more than 7°C within 1 h and lasted for 4.5 h, mortality was 100%. However, no mortality was observed when THI was elevated from 24°C to 8°C over a period of 3 h. When THI reached from 25.2° to 33.9°C in 4 h after the beginning of heat stress, 29.2% mortality was observed. The current results show that, although there was a similarity in temperature and relative humidity values both at the initiation and conclusion of the thermal challenge between experiments, mortality was much more severe in the hens exposed to a thermal environment with a sharp elevation in THI as compared with mortality when THI changed moderately (Figures 1, 2). The severe effects of acute heat stress were also evident when we observed the responses of hens experiencing a thermal environment wherein THI was gradually elevated to a plateau that was higher and lasted longer than that in Experiment 1 (Figure 3). These results suggest that an acute elevation in THI is remarkably detrimental to hens.

One of the most detrimental effects of heat stress is mortality, which is easy to assess on the spot at farms when birds are exposed to severe thermal environments. Nevertheless, since there is neither a standardized method to record mortality nor accurate records of age or causes of mortality, it is difficult to summarize the available data on mortality in chronic heat stress (14). Mortality was remarkably increased in laying hens exposed to a constantly high temperature for 5 weeks (31.7%), exceeding those for the cyclic heat stress (6.7%) or control (5%) groups (3).

Recently, the number of mortalities in livestock has been increasing due to heat stress in Korea. The majority of mortalities occur in chickens, and are markedly increased during the heat wave that follows the rainy season in Korea (26, 27), a period when the ambient temperature suddenly increases with high humidity. It has also been shown that acute heat stress had a more severe effect on mortality than did chronic heat stress, when broilers were exposed for 10 consecutive days to one of three thermal conditions (control: 20°C; chronic heat stress: 30 ± 2°C; or acute heat stress: 35 ± 2°C, 4 h/d), and that heat stress itself is a risk factor for impaired intestinal integrity resulting in increased intestinal permeability to endotoxins and bacterial contamination (28). These authors also reported differential responses in corticosterone, cytokine, and salmonella prevalence in tissues between chronic and acute heat stress in broilers.

THI is an index that considers the combined effects of both temperature and relative humidity rather than considering each separately. When individual effects were tested, however, they appear to differentially affect hens' performance (29): changes in relative humidity (40 vs. 60%) had stronger effects on hen-day egg production and mortality than did changes in temperature (23.9° vs. 31.1°C). In addition, hens in higher relative humidity (60%) had higher blood pH and mortality, but lower pCO_2_ and ${\text{HCO}}_{3}^{-}$ than those in lower relative humidity (40%) although body temperature remained the same. Similar results were also observed with higher ambient temperature (23.9° vs. 31.1°C) which resulted in lower pCO_2_ and ${\text{HCO}}_{3}^{-}$ without affecting body temperature and mortality (29).

There was a consistent egg-laying performance under THI of 27.5, above which hens' performance started to decline (30). In 40-day-old broilers, THI of 28°C was considered a reasonable threshold for the onset of heat stress (31), whereas a THI of 30.6°C was suggested for broilers older than 31 days due to higher mortality above this temperature (19). Broilers between 31 and 40 days of age showed increased mortality when the maximum THI and temperature of the day were above 30.6 and 34.4°C, respectively (19).

Panting is one of the most important ways by which poultry give off heat as they do not have sweat glands, and it is also affected by the environment wherein both ambient temperature and relative humidity are two key factors. In the current study, panting-like behavior was not observed in any of the hens under THI of 25°C, but was found in 40% birds at 28.5°C, and in 100% above 31°C. Although hens were placed in similar thermal environments, a longer exposure time caused higher panting rates and mortality. Furthermore, panting frequency was largely variable in hens under THI of 30°C. This observation is in accordance with others. The panting threshold was extremely variable even when hens were of the same age and breeding, and were acclimatized to constant temperatures (32). In laying hens experiencing ambient temperatures of 32, 35, 38, or 41°C, panting was not observed at 32°C, but did occur at 35°C with slight alkalosis (pH 7.55) without differences in rectal temperature (24). However, panting was observed at 38°C with an increased rectal temperature and moderate alkalosis (pH 7.58), and further panting was observed at 41°C with severe alkalosis (pH 7.65) and considerably high rectal temperature. Respiratory rates increased from 74 at 0 h, 218 at 1 h, and to 207 at 4 h in 42-day-old broilers kept at an ambient condition of 36°C and 60% relative humidity (33). Similar findings were reported in egg-laying ducks exposed to chronic heat stress, which led to increases in panting rates and spreading of wings and reduction in feed intake, laying rates and egg quality, including egg weight, eggshell thickness and strength, and Haugh unit (34).

In the current study, heat stress significantly reduced plasma albumin, amylase, and AST, but not for the rest of the parameters measured, including corticosterone. A 2-h heat stress at 32°C did not have a significant effect on plasma corticosterone, glucose, and uric acid in laying hens before and after the stress (35). However, a long-term heat stress for 16 weeks reduced plasma corticosterone small but significantly and this result was influenced by age (36). In a study in which 68-week-old Lohmann Brown-Lite hens were exposed either to 25°C as a control or to high ambient temperatures gradually rising from 25° to 33°C from 11:00 to 18:00 h for 28 days, Borzouie et al. (37) showed that heat stress resulted in a significant reduction in plasma protein (24.3%) and globulin (33.1%) while significantly increasing cholesterol (164.3%), alanine aminotransferase (300%), and glutamate dehydrogenase (885.2%). However, there were no significant changes in albumin, AST, glucose, alkaline phosphatase, γ-glutamyltransferase, and heterophil/lymphocyte ratios. Increased plasma concentrations in albumin, amylase, and AST may be associated with stress conditions such as dietary corticosterone (25). Together, these results suggest that blood data from stress studies should be cautiously interpreted.

Although 70-week-old laying hens were used in the current study to determine the effects of THI on mortality and panting, the effects could be different depending on age, genotype, and group size of hens (1, 14). Age and genotype in laying hens are important aspects for resilience to heat stress as both factors are strong contributors to variations in egg production and quality. In broiler breeds, younger birds could be more resilient to higher thermal environments than older ones. Cloacal temperature during daytime was age-dependently higher in broilers (38). Vale et al. (19) reported that there was no impact of heat waves in broilers aged between 29 and 31 days, but increased mortality was found in broilers older than 31 days when the maximum THI was above 30.6°C, the main feature of days presenting heat wave. There are differential effects of heat stress in three strains of laying hens (1). Hy-Line Brown hens were used in this study, but the responses could be different depending on strains. Franco-Jimenez et al. (1) investigated differential effects of heat stress in Hy-Line Brown, W36, and W98 hens that were exposed to heat stress at 35°C for 2 weeks, followed by a 2-week recovery period at 22°C, and found that mortality rates were 16, 8, and 4% for the Brown, W98, and W36 hens, respectively. In contrast, their production parameters (egg production, and egg yolk, albumen, and shell weights) were different, with the Hy-Line W98 hens tolerating better heat stress than did the Brown or W36 hens.

In the current study, feed intake, water intake, egg production, egg quality, and body weight were not measured since the primary goal was to determine mortality. These parameters are largely affected in laying hens experiencing heat stress, depending upon the severity and duration of the thermal challenge (1, 3, 39). One study in which laying and non-laying hens of the same age and strain were exposed to temperatures up to 48.3°C reported that weight and laying may not be the main factors influencing the hen's response to heat stress (40).

The results from the current study clearly show the inability of laying hens to tolerate thermal environments in which THI was rapidly elevated to high levels, which has never been addressed so far. Our research suggests that, the faster and higher the increase in THI, the more serious mortality will be in heat-stressed laying hens. By using THI, which considers both ambient temperature and humidity, the impact of heat stress in laying hens can be compared among different settings of various studies. Moreover, this study suggests the importance of incorporating reliable tools to slow or monitor temperature rises (e.g., THI) within the poultry house, which has practical relevance to the global poultry industry.

## Data Availability Statement

All datasets presented in this study are included in the article.

## Ethics Statement

The animal study was reviewed and approved by Institutional Animal Care and Use Committee Konkuk University.

## Author Contributions

Y-HC, H-HC, and K-WL conceived and designed the study. Y-HC and SK wrote the paper with a critical review by all authors and analyzed the data. SK, D-HK, SL, and TL conducted the experiments. All authors contributed to the article and approved the submitted version.

## Conflict of Interest

BM was employed by the company Celltech Co., Ltd. The remaining authors declare that the research was conducted in the absence of any commercial or financial relationships that could be construed as a potential conflict of interest.
